# MicroRNA-150 Deletion from Adult Myofibroblasts Augments Maladaptive Cardiac Remodeling Following Chronic Myocardial Infarction

**DOI:** 10.3390/biom14121650

**Published:** 2024-12-22

**Authors:** Satoshi Kawaguchi, Marisa N. Sepúlveda, Jian-peng Teoh, Taiki Hayasaka, Bruno Moukette, Tatsuya Aonuma, Hyun Cheol Roh, Meena S. Madhur, Il-man Kim

**Affiliations:** 1 Department of Anatomy, Cell Biology, and Physiology, Indiana University School of Medicine, Indianapolis, IN 46202, USA; s-kawa@asahikawa-med.ac.jp (S.K.); masepulv@iu.edu (M.N.S.); jpteoh146017@gmail.com (J.-p.T.); taikih@asahikawa-med.ac.jp (T.H.); bruno.moukette@pfizer.com (B.M.); aonu@asahikawa-med.ac.jp (T.A.); 2 Department of Emergency Medicine, Asahikawa Medical University, Asahikawa 078-8510, Hokkaido, Japan; 3 Division of Cardiology and Nephrology, Department of Internal Medicine, Asahikawa Medical University, Asahikawa 078-8510, Hokkaido, Japan; 4 Internal Medicine Research Unit, Pfizer Inc., Cambridge, MA 02139, USA; 5 Department of Biochemistry and Molecular Biology, Indiana University School of Medicine, Indianapolis, IN 46202, USA; hyunroh@iu.edu; 6 Division of Clinical Pharmacology, Indiana University School of Medicine, Indianapolis, IN 46202, USA; mmadhur@iu.edu; 7 Krannert Cardiovascular Research Center, Indiana University School of Medicine, Indianapolis, IN 46202, USA

**Keywords:** cardiac remodeling, heart failure, microRNAs, myocardial infarction, myofibroblast gene regulation, profibrotic genes

## Abstract

MicroRNA (miR: small noncoding RNA)-150 is evolutionarily conserved and is downregulated in patients with diverse forms of heart failure (HF) and in multiple mouse models of HF. Moreover, miR-150 is markedly correlated with the outcome of patients with HF. We previously reported that systemic or cardiomyocyte-derived miR-150 in mice elicited myocardial protection through the inhibition of cardiomyocyte death, without affecting neovascularization and T cell infiltration. Our mechanistic studies also showed that the protective roles of miR-150 in ischemic mouse hearts and human cardiac fibroblasts were, in part, attributed to the inhibition of fibroblast activation via the repression of multiple profibrotic genes. However, the extent to which miR-150 expression in adult myofibroblasts (MFs) modulates the response to myocardial infarction (MI) remains unknown. Here, we develop a novel 4-hydroxytamoxifen-inducible MF-specific miR-150 conditional knockout mouse model and demonstrate that the mouse line exhibits worse cardiac dysfunction after MI. Our studies further reveal that miR-150 ablation selectively in adult MFs exacerbates cardiac damage and apoptosis after chronic MI. Lastly, MF-specific miR-150 deletion in adult mice promotes the expression of proinflammatory and profibrotic genes as well as cardiac fibrosis following chronic MI. Our findings indicate a key protective role for MF-derived miR-150 in modulating post-MI responses.

## 1. Introduction

The process of prolonged cardiac fibrosis, characterized by excessive extracellular matrix deposition, is a critical factor in the development of heart failure (HF). This fibrotic response is primarily driven by activation of cardiac fibroblasts (CFs) into myofibroblasts (MFs) in response to injury or stress [1,2]. Although the significance of CF activation in HF has been established, our understanding of the underlying mechanisms involved in this process remains incomplete.

MicroRNAs (miRNAs or miRs) are a class of small noncoding RNAs approximately 22 nucleotides in length that play critical roles in post-transcriptional regulation by repressing target mRNAs. Studies investigating gain- and loss-of-function studies of various miRs have revealed their involvement in the pathogenesis of various heart diseases [3,4], indicating their potential as therapeutic targets for conditions such as cardiac fibrosis [5]. Manipulating miR biogenesis has emerged as a crucial underlying mechanism of HF [6,7,8,9,10], with new miR therapies being explored in clinical trials [11].

In previous research, we observed that miR-150 expression was activated by the β-blocker, carvedilol, through β-arrestin-mediated β_1_-adrenergic receptor (β_1_AR) protective signaling [12]. Using knockout (KO) mouse models, we also demonstrated that β_1_AR/β-arrestin-responsive miR-150 played a beneficial role in post-myocardial infarction (MI) remodeling [13,14]. Additionally, cardiac-specific overexpression (O/E) of miR-150 blunted HF induced by transverse aortic constriction (TAC) [15]. Loss of miR-150 resulted in increased cardiac fibrosis following TAC, with miR-150 being decreased specifically in CFs rather than cardiomyocytes (CMs) isolated from mice subjected to TAC [16]. Furthermore, miR-150 was found to inhibit CF activation in vitro [16]. Our mechanistic studies also revealed that the beneficial effects of miR-150 on ischemic hearts or human CFs were attributed to the inhibition of fibroblast activation (e.g., fibroblast proliferation, fibroblast migration, and MF accumulation) via its repression of various profibrotic genes [17,18]. In addition, our research demonstrated that O/E of miR-150 attenuated cardiac fibrosis and adverse remodeling following MI induced by a profibrotic long noncoding RNA called MIAT [17]. Furthermore, we found that miR-150 improved cardiac function and reduced fibrosis in mice with cardiac-specific O/E of β_1_ARs that lack the ability to activate β-arrestins (cardiac-specific GRK–β_1_AR transgenic mice [19]) after prolonged catecholamine stimulation [20]. These findings suggest that miR-150 derived from MFs could be a crucial downstream target through which β_1_AR/β-arrestin signaling inhibits CF activation and promotes beneficial remodeling in HF.

Interestingly, miR-150 has been found to be significantly decreased in patients with various cardiovascular diseases [21,22,23,24] and in distinct mouse models of HF [13,15,25]. Moreover, miR-150 is highly conserved across species and is markedly associated with HF outcomes in humans [26]. These findings underscore the potential clinical implications of miR-150 regulatory mechanisms in HF. However, there is a lack of definitive studies using appropriate mouse models to address cell type-specific actions of miR-150. Specifically, the role of miR-150 expressed selectively in adult MFs in regulating the response to MI remains to be elucidated.

In the current study, we utilize a novel inducible MF-specific miR-150 conditional KO (cKO) mouse model. We demonstrate that the deletion of miR-150 specifically in MFs of adult mice exacerbates cardiac dysfunction as well as cardiac damage, apoptosis, and fibrosis following chronic MI. These findings provide direct evidence that MF is a primary cell type responsible for the beneficial effects mediated by miR-150 post-MI. Our results highlight the crucial role of MF-derived miR-150 as a key regulator of fibrogenesis, cardiac fibrosis, and ischemic HF.

## 2. Materials and Methods

### 2.1. Availability of Data and Materials

All data are included in the manuscript. For the purposes of reproducing the results or replicating the procedures, all methods and study reagents will be available to other researchers upon reasonable requests.

### 2.2. 4-Hydroxytamoxifen-Inducible Myofibroblast-Restricted Knockout of miR-150 in Mice

To establish a novel inducible MF-restricted miR-150 cKO mouse model, we bred our previously reported miR-150 ^flox/flox^ (miR-150 ^fl/fl^) mice [14] with Postn-MerCreMer/+ mice (JAX, 029645) [27]. The Postn-MerCreMer mice expressed tamoxifen-inducible Cre recombinase from the promoter of the mouse *Postn*, which was reported to be expressed almost exclusively in activated MFs after injury [27,28,29]. The miR-150 ^fl/+^;Postn-MerCreMer mice were subsequently crossed back to miR-150 ^fl/fl^ mice to obtain the inducible MF-specific miR-150 cKO mice (miR-150 ^fl/fl^; Postn-MerCreMer). All mice were maintained on a C57BL/6J background, and genetically matched Cre-negative miR-150 ^fl/fl^ littermates were used as controls. We then injected 4-hydroxytamoxifen (4-OH-TAM; 20 mg/kg/day, intraperitoneally [I.P.] for 5 days) in adult mice as established previously [30]. Administration of 4-OH-TAM was initiated immediately after MI, as previously validated for Postn-MerCreMer/+ mice that did not show 4-OH-TAM-induced cardiotoxicity even after injury [27,28,29]. Genotyping for Postn-MerCreMer heterozygous mice was done using primers (5′-GGTGGGACATTTGAGTTGCT-3′ and 5′-CCTTGCAATAAGTAAAACAGCTC-3′) to amplify a 270 bp gene product specific for the transgene gene. Genotyping for miR-150 floxed mice was done with the primers of 5′-GAAGGGTTCCTGTCCTTGTTGGC-3′ and 5′-AGTAAGGGTGGAGCCTCTGACCT-3′, resulting in band sizes of 250 bp for the wild-type allele and 300 bp for the floxed allele.

### 2.3. Mouse Model of Myocardial Infarction and Post-Myocardial Infarction Mortality

Eight–16-week-old miR-150 cKO or miR-150 ^fl/fl^ mice were subjected to MI as we published [13,17,18,31]. In brief, mice were anesthetized using isoflurane (1–4%, inhalant) and placed on a heating pad. Mice were intubated and ventilated with oxygen using a PhysioSuite MouseVent ^TM^ ventilator (Kent Scientific Corporation, Torrington, CT, USA). The left anterior descending (LAD) coronary artery was visualized under a stereoscope and ligated by using an 8-0 nylon suture. Regional ischemia was visually confirmed by discoloration of the occluded distal myocardium. Sham-operated mice underwent the same procedure without LAD occlusion. The topical local analgesia drug bupivacaine (a few small drops of 0.75–1%) was administered at the time of surgery. Sustained-release meloxicam (4–5 mg/kg, subcutaneous) and sustained-release buprenorphine (3.25 mg/kg Ethiqa XR; MWI Animal Health, Boise, USA, subcutaneous) were also given once to provide up to 72 h of systemic analgesia. The mice were observed for the pinch-toe reflex every 15 min during the surgery. Following the surgery, the mice were monitored until they regained consciousness. Post-operative care included monitoring every 15–30 min following the surgery for 2–3 h and then daily until the study endpoint for signs of distress, including difficulty with breathing, grooming, defecation, eating, and mobility. The responses to toe/skin pinch and heart rate were used for the optimal anesthesia and appropriate post-operative monitoring plan. Mice whose pain could not be managed and who exhibited such symptoms of distress were euthanized immediately and humanely. Records detailing the procedures and pharmacological interventions given to the mice were maintained. We also monitored the survival of the 4-OH-TAM-inducible miR-150 cKO mice and their miR-150 ^fl/fl^ control littermates following MI.

### 2.4. Echocardiographic Assessment of Left Ventricular Function

Left ventricular performance was assessed by two-dimensional transthoracic high-resolution echocardiography using a Vevo 2100 Ultrasound (FUJIFILM VisualSonics, Inc., Toronto, ON, Canada) at pre-surgery (baseline) and post-MI (1, 2, and 4 weeks) as we published [31]. The M-mode tracings were used to measure left ventricular performance. The parameters, which included left ventricular internal diameter (LVID) in either diastole (LVIDd) or systole (LVIDs), end-diastolic volume (EDV), and end-systolic volume (ESV), were obtained. Echocardiography and data analysis were performed by a single operator blinded to the mouse genotypes. The fractional shortening (FS) was calculated according to the following formula: FS (%) = [(LVIDd-LVIDs)/LVIDd] × 100. The ejection fraction (EF) was calculated by: EF (%) = [(EDV-ESV)/EDV] × 100.

### 2.5. Morphological, Histopathological, and Immunohistochemical Analyses

Morphometric analyses of the mouse hearts were carried out as previously published [13,31]. Histopathological analyses of the hearts, such as fibrosis via Masson’s Trichrome or Picro Sirius Red staining, were conducted as we described in previous publications [17,18]. Heart sections were stained with hematoxylin and eosin (H&E) for gross histological assessment. Cardiac sections were also stained for cleaved-caspase 3 using the Dako Autostainer Link 48 to assess apoptosis as we published [14]. Briefly, cardiac sections were deparaffinized, rehydrated, and subjected to an EDTA antigen retrieval for 5 min in a pressure cooker, endogenous enzyme block for 15 min, primary antibody incubation (cleaved-caspase 3, rabbit polyclonal [1:200 dilution, 9661, Cell Signaling]) for 60 min, and Dako EnVision-HRP reagent incubation for 30 min. Signals were detected by adding hydrogen peroxide using diaminobenzidine as a chromogen, followed by hematoxylin counterstaining. Brown cells were quantified as number of positive cells × 100/total cells in six random microscopic (20×) fields in each slide. All histology images presented in this study are from the same field in serial sections. Digital photographs of staining were captured with a Keyence (Osaka, Japan) microscope (BZ-X810) and processed with Adobe Photoshop 2024.

### 2.6. RNA Isolation and Quantitative Real-Time Reverse-Transcription PCR (QRT-PCR)

The hearts were excised, flash frozen in liquid nitrogen, and subjected to RNA isolation and QRT-PCR analyses. Total RNAs from the infarct area of mouse hearts were then prepared using TRIzol Reagent (Thermo Fisher Scientific, Waltham, MA, USA) and treated with RNase-free DNase I (Thermo Fisher Scientific, Waltham, MA, USA) as we previously published [32]. For measuring the expression of mature miR-150, the TaqMan MicroRNA Reverse Transcription Kit (a highly specific kit that generates only mature miRs, not precursors; Thermo Fisher Scientific, Waltham, MA, USA) was used to generate cDNAs. We used the miR-150 TaqMan probe (000473; Thermo Fisher Scientific, Waltham, MA, USA) to measure the mature miR-150 by QRT-PCR. A U6 snRNA probe (001973; Thermo Fisher Scientific, Waltham, MA, USA) was used for an endogenous control. The cDNAs for gene expression analyses were generated using SuperScript IV reverse transcriptase (Thermo Fisher Scientific, Waltham, MA, USA) and random hexamers. Gene expression was detected using TaqMan gene expression assays for mouse (*Nppa*, Mm01255747_g1; *Nppb*, Mm01255770_g1; *Acta1*, Mm00808218_g1; *Il-6*, Mm00446190_m1; *Il-1b*, Mm00434228_m1; *Bak1*, Mm00432045_m1; *Egr2*, Mm00456650_m1; *Ctgf*, Mm01192933_g1; *Snail*, Mm00441533_g1; *Runx3*, Mm00490666_m1; *Sprr1a*, Mm01962902_s1; and *Gapdh*, Mm99999915_g1 for an endogenous control). QRT-PCR reactions were analyzed using a QuantStudio 3 Detection System (Thermo Fisher Scientific, Waltham, MA, USA), as we have previously published [32]. The expression compared to endogenous controls was calculated using 2^−ΔΔCt^, and the expression levels were normalized to control.

### 2.7. Statistical Analysis

Data are reported as mean ± SEM (except serial echocardiographic data, in which we used SD because no clear variation bars are shown otherwise) from independent experiments with different biological samples per group. Most graphical data are presented as scatter/dot plots to allow the direct evaluation of the distribution of the data. We assessed normality by the Kolmogorov–Smirnov test. Statistical significance was determined by unpaired two-tailed *t*-tests for comparisons between two groups, one-way ANOVA with Tukey’s multiple comparison test for multiple groups, and two-way ANOVA with Tukey’s multiple comparison test for comparisons between two groups with different conditions. N was 3–11, and we present the exact sample size for each experimental group/condition as a number in the figure legend. A *p* value of <0.05 was considered statistically significant. We indicate *p* values as follows: * or ^#^ *p* < 0.05; ** or ^##^ *p* < 0.01; and *** or ^###^ *p* < 0.001.

### 2.8. Ethics Committee Approval for Animal Study

The use of animals in this study was conformed to the Guide for the Care and Use of Laboratory Animals published by the US National Institutes of Health. The mice were euthanized by asphyxiation with CO_2_ to minimize the pain and time needed for cessation of life. The secondary method of euthanasia (bilateral thoracotomy, cervical dislocation, exsanguination, or decapitation) was then applied under 1–4% inhalant isoflurane. These methods are consistent with the recommendations of the Panel on Euthanasia of the American Veterinary Medical Association. We performed all experiments with mice according to the protocols approved by the Institutional Animal Care and Use Committee at the Indiana University (approval reference #21189). We used 8–16-week-old C57BL/6J mice of both sexes for the current study. We randomly assigned genotype- and sex-matched mice to experimental groups to mitigate the cage effect. We masked the genotypes of the animals for researchers until the end of the analysis.

## 3. Results

### 3.1. MiR-150 Selectively in Adult Myofibroblasts Ameliorates Cardiac Dysfunction After Myocardial Infarction

**Figure 1:**
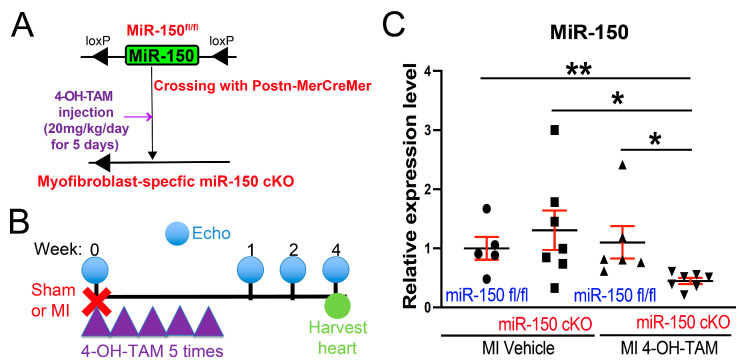
Establishment of a novel inducible myofibroblast-specific miR-150 knockout mouse line. (**A**) Targeting scheme, mouse crossing, and establishment of 4-hydroxytamoxifen (4-OH-TAM)-inducible myofibroblast (MF)-restricted conditional knockout (cKO) of miR-150 in vivo. (**B**) Experimental timeline with myocardial infarction (MI) and 4-OH-TAM injection (20 mg/kg/day for 5 days) followed by echocardiography and harvest. 4-OH-TAM injection was initiated immediately after MI. (**C**) QRT-PCR analyses of miR-150 in left ventricles from miR-150 ^fl/fl^ or miR-150 cKO mice that were intraperitoneally injected with vehicle or 4-OH-TAM. Left ventricles were harvested at 4 weeks after MI. N = 5–7 per group. Data are presented as mean ± SEM. One-way ANOVA with Turkey multiple comparison test. * *p* < 0.05 or ** *p* < 0.01 vs. 4-OH-TAM-inducuble MF-restricted miR-150 cKO mice.

We previously demonstrated that miR-150 functioned as a key regulator in preventing human CF activation by inhibiting multiple profibrotic genes [17,18]. Additionally, miR-150 negatively regulated mouse CF activation in vitro [16]. However, the role of MF-derived miR-150 in the heart in vivo remained unclear. To address this gap, we generated a novel inducible MF-restricted miR-150 cKO mouse line using 4-OH-TAM (Figure 1A). Mice at 8–16 weeks of age were first subjected to MI and injected with 4-OH-TAM for 5 days post-surgery. The administration of 4-OH-TAM began immediately after MI induction, as previously described [27,28]. Based on an excessive fibrotic response over the first week after MI induction, we performed serial echocardiography at 1–4 weeks post-MI and harvested hearts at 4 weeks post-MI (Figure 1B). By breeding miR-150 ^fl/fl^ mice [14] with Postn-MerCreMer mice [27] and administering 4-OH-TAM (20 mg/kg/day, I.P. for 5 days) to adult mice as validated previously [30], we were for the first time able to successfully demonstrate the significant downregulation of miR-150 expression in the left ventricles of the inducible miR-150 cKO MI mice compared to three control groups (Figure 1C).

**Figure 2:**
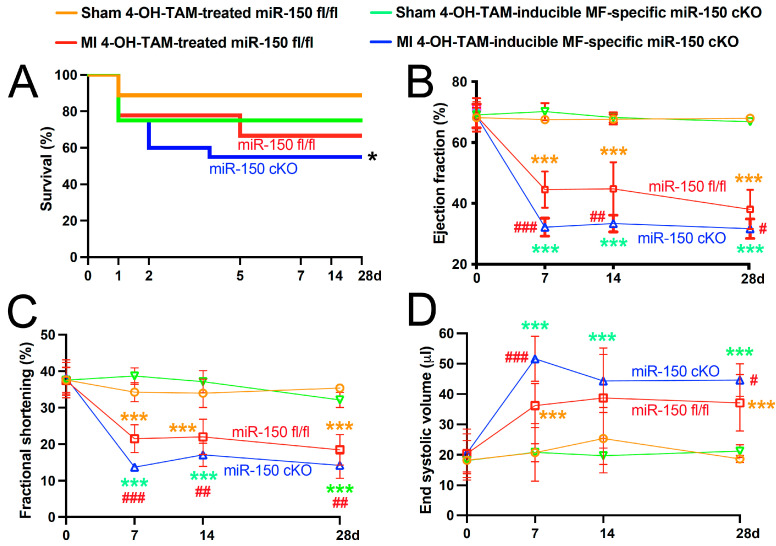
Inducible myofibroblast-restricted miR-150 deletion in adult mice augments cardiac dysfunction after myocardial infarction. (**A**) Kaplan–Meier survival curve at 0–28 days (d) following myocardial infarction (MI) in 4-OH-TAM-inducible miR-150 cKO mice or 4-OH-TAM-treated miR-150 ^fl/fl^ littermates. N = 3–11 per group. Log-rank test. * *p* < 0.05 vs. other three groups. (**B**–**D**), Transthoracic echocardiography was conducted on the four experimental groups (sham and MI of 4-OH-TAM-treated miR-150 ^fl/fl^ and 4-OH-TAM-inducible miR-150 cKO) at 0–28 days (d) post-MI. Quantification of left ventricular (LV) ejection fraction (EF: (**B**)), fractional shortening (FS: (**C**)), and end-systolic volume (LVESV: (**D**)) is shown. N = 3–11 per group. Data are presented as mean ± SD. Two-way ANOVA with Tukey’s multiple comparison test. *** *p* < 0.001 vs. Sham of same genotype (denoted by two different colors for sham within same group); ^#^ *p* < 0.05, ^##^ *p* < 0.01, or ^###^ *p* < 0.001 vs. MI 4-OH-TAM-treated miR-150 ^fl/fl^ (denoted by red).

Subsequently, we evaluated post-MI cardiac function in four groups: sham and MI groups of miR-150 ^fl/fl^ and miR-150 cKO mice treated with 4-OH-TAM. As expected, we found that the inducible MF-restricted miR-150 cKO mice displayed normal cardiac function at baseline (Figure 2B–D).

Despite exhibiting normal cardiac function at baseline, the 4-OH-TAM-inducible miR-150 cKO mice responded differently to MI induced by permanent ligation of the LAD. The MI resulted in a significant increase in mortality in 4-OH-TAM-inducible miR-150 cKO mice compared with the miR-150 ^fl/fl^ littermate controls (Figure 2A), like systemic miR-150 KO mice that showed increased mortality due to an augmented inflammatory response and cardiac rupture following acute MI [13]. At 1 week post-MI, the 4-OH-TAM-inducible miR-150 cKO mice showed a significant deterioration in cardiac function, evidenced by decreased EF and FS, along with increased ESV (Figure 2B–D). We also found that compared to the 4-OH-TAM-treated miR-150 ^fl/fl^ controls, the 4-OH-TAM-inducible miR-150 cKO mice displayed a significant decrease in cardiac output (13.33 ± 0.61 vs. 17.09 ± 1.05, two-way ANOVA with Tukey’s multiple comparison test, ** *p* < 0.01) and left ventricular posterior wall thickness in systole (0.89 ± 0.04 vs. 1.05 ± 0.05, two-way ANOVA with Tukey’s multiple comparison test, * *p* < 0.05), coupled with increased EDV (84.65 ± 2.40 vs. 65.70 ± 4.93, two-way ANOVA with Tukey’s multiple comparison test, ** *p* < 0.01) at 1 week after MI.

This LV functional impairment in 4-OH-TAM-inducible miR-150 cKO mice persists at 2 weeks post-MI, characterized by decreased EF and FS (Figure 2B–D). The cardiac dysfunction further worsens at 4 weeks after MI, with significant decreases in EF and FS, accompanied by significant increases in ESV (Figure 2B–D) and EDV (82.08 ± 3.55 vs. 61.08 ± 4.76, two-way ANOVA with Tukey’s multiple comparison test, ** *p* < 0.01). In contrast, 4-OH-TAM-treated miR-150 ^fl/fl^ control mice exhibit less cardiac functional impairment at all time points following MI (Figure 2B–D). The inducible MF-restricted miR-150 cKO mice thus display augmented cardiac dysfunction post-MI compared to the three control groups (Figure 2), similar to findings observed in systemic miR-150 KO mice [13]. These data collectively suggest that the selective deletion of miR-150 in adult MFs is sufficient to exacerbate cardiac dysfunction post-MI.

### 3.2. Myofibroblast-Restricted miR-150 Deletion Augments Damage and Apoptosis as Well as the Expression of Inflammatory Genes in the Heart After Chronic Myocardial Infarction

**Figure 3:**
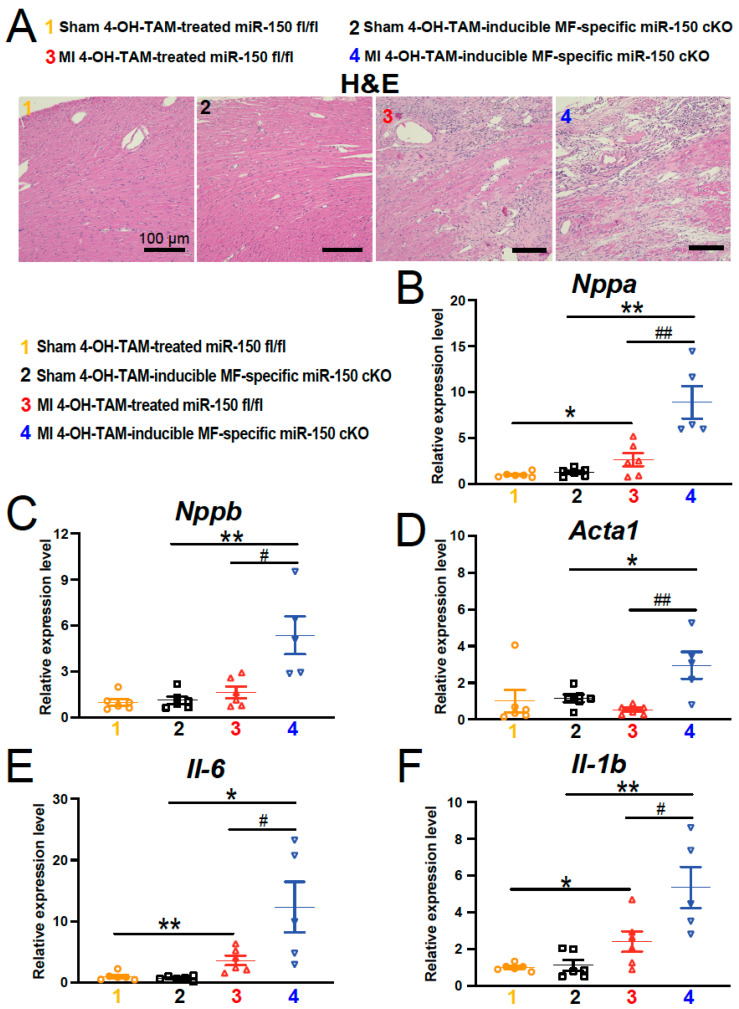
Selective deletion of miR-150 in adult myofibroblasts induces damage and the expression of inflammatory genes in the heart after chronic myocardial infarction. (**A**), Representative hematoxylin and eosin (H&E) staining of heart sections of the peri-ischemic border area at 4 weeks after MI reveals increased disorganized structure in 4-OH-TAM-inducible miR-150 cKO hearts compared to 4-OH-TAM-treated miR-150 ^fl/fl^ controls. Scale bars: 100 μm. (**B**–**D**) QRT-PCR analysis of *Nppa*, *Nppb*, and *Acta1* expression representing cardiac damage in ischemic areas from 4-OH-TAM-inducible miR-150 cKO hearts compared to 4-OH-TAM-miR-150 ^fl/fl^ controls at 4 weeks after MI. (**E**,**F**), QRT-PCR analysis of *Il-6* and *Il-1b* expression for cardiac inflammation in ischemic areas from 4-OH-TAM-inducible miR-150 cKO hearts compared to 4-OH-TAM-treated miR-150 ^fl/fl^ controls at 4 weeks after MI. N = 5–6 per group. QRT-PCR data are presented as fold induction of gene expression normalized to *Gapdh*. Data are shown as mean ± SEM. Two-way ANOVA with Tukey’s multiple comparison test. * *p* < 0.05 or ** *p* < 0.01 vs. sham of same genotype; ^#^ *p* < 0.05 or ^##^ *p* < 0.01 vs. MI 4-OH-TAM-treated miR-150 ^fl/fl^.

In our previous study, we demonstrated that systemic miR-150 KO mouse hearts exhibited excessive damage, neutrophil infiltration, and CM apoptosis during acute MI, without affecting neovascularization and T cell infiltration [13]. To assess whether the ablation of miR-150 specifically from adult mouse MFs leads to maladaptive remodeling after MI, we utilized 4-OH-TAM-inducible miR-150 cKO mice and compared the post-MI remodeling in these mice with that of 4-OH-TAM-treated miR-150 ^fl/fl^ controls. We observed that hearts from the 4-OH-TAM-inducible miR-150 cKO mice displayed greater accumulation of additional nuclei, necrosis, and disorganized structure compared to hearts from 4-OH-TAM-treated miR-150 ^fl/fl^ MI mice, as evidenced by histological analysis at 4 weeks post-MI (Figure 3A). Consistently, mRNA levels of the fetal genes *Nppa*, *Nppb*, and *Acta1* were also significantly elevated in 4-OH-TAM-inducible miR-150 cKO hearts after 4 weeks of MI (Figure 3B–D). Furthermore, we investigated the inflammatory response in the hearts of 4-OH-TAM-inducible miR-150 cKO mice at 4 weeks after MI. We found increased expression of inflammatory *Il-6* and *Il-1b* in these hearts compared to the 4-OH-TAM-treated miR-150 ^fl/fl^ controls after chronic MI (Figure 3E,F), similar to our observations in systemic miR-150 KO mice [13].

**Figure 4:**
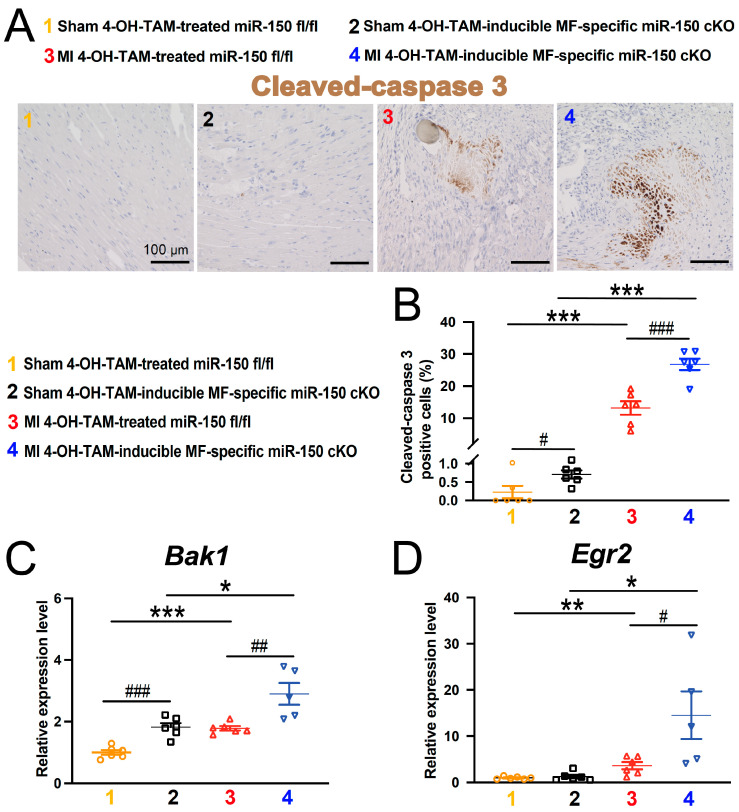
Inducible myofibroblast-specific miR-150 loss in adult mice exacerbates apoptosis in the heart following chronic myocardial infarction. (**A**,**B**) Representative cleaved-caspase 3 staining images in heart sections of the peri-ischemic border area at 4 weeks after MI (**A**) and quantification of apoptosis in six 20× fields (**B**). Scale bars: 100 μm. (**C**,**D**) QRT-PCR analysis of proapoptotic *Bak1* and *Egr2* expression in the ischemic areas from 4-OH-TAM-inducible miR-150 cKO hearts compared to 4-OH-TAM-treated miR-150 ^fl/fl^ controls at 4 weeks after MI. QRT-PCR data are presented as fold induction of gene expression normalized to *Gapdh*. N = 5–6 per group. Data are shown as mean ± SEM. Two-way ANOVA with Tukey’s multiple comparison test. * *p* < 0.05, ** *p* < 0.01, or *** *p* < 0.001 vs. sham of same genotype; ^#^ *p* < 0.05, ^##^ *p* < 0.01, or ^###^ *p* < 0.001 vs. 4-OH-TAM-treated miR-150 ^fl/fl^.

Additionally, apoptosis was quantified in the hearts at 4 weeks after MI using cleaved-caspase 3 staining. The 4-OH-TAM-inducible miR-150 cKO hearts exhibited significantly higher numbers of cleaved-caspase 3-positive cells in the infarct and border regions compared to the 4-OH-TAM-treated miR-150 ^fl/fl^ hearts after chronic MI (Figure 4A,B). Moreover, the expression of apoptotic *Bak1* and *Egr2* was elevated in 4-OH-TAM-inducible miR-150 cKO hearts compared to controls at 4 weeks after MI (Figure 4C,D). Overall, these findings suggest that MF-derived miR-150 plays a protective role against murine MI by regulating cardiac remodeling post-MI.

### 3.3. Myofibroblast-Specific Ablation of miR-150 in Adult Mice Exacerbates Cardiac Fibrosis Following Chronic Myocardial Infarction

**Figure 5:**
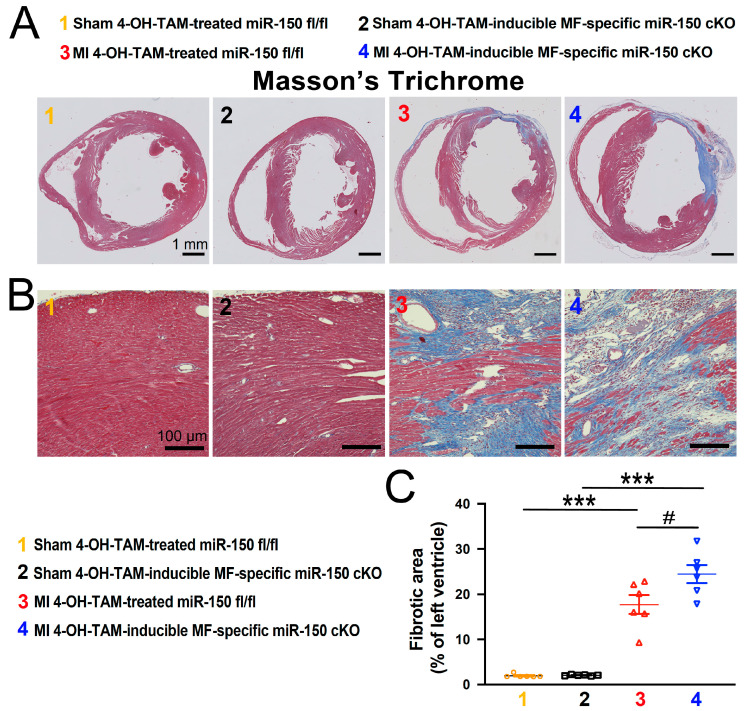
Selective ablation of miR-150 in adult myofibroblasts promotes cardiac fibrosis after chronic myocardial infarction. Representative Masson’s Trichrome staining (**A**,**B**) in heart sections from the four experimental groups at 4 weeks after MI and fibrosis quantification (**C**) in whole left ventricles. Fibrosis histology images from whole heart longitudinal sections ((**A**): Scale bars: 1 mm) and zoomed in images of the peri-ischemic border area ((**B**): Scale bars: 100 μm). N = 5–6 per group. Data are shown as the mean ± SEM. Two-way ANOVA with Tukey’s multiple comparison test. *** *p* < 0.001 vs. sham of same genotype; ^#^ *p* < 0.05 vs. MI 4-OH-TAM-treated miR-150 ^fl/fl^.

**Figure 6:**
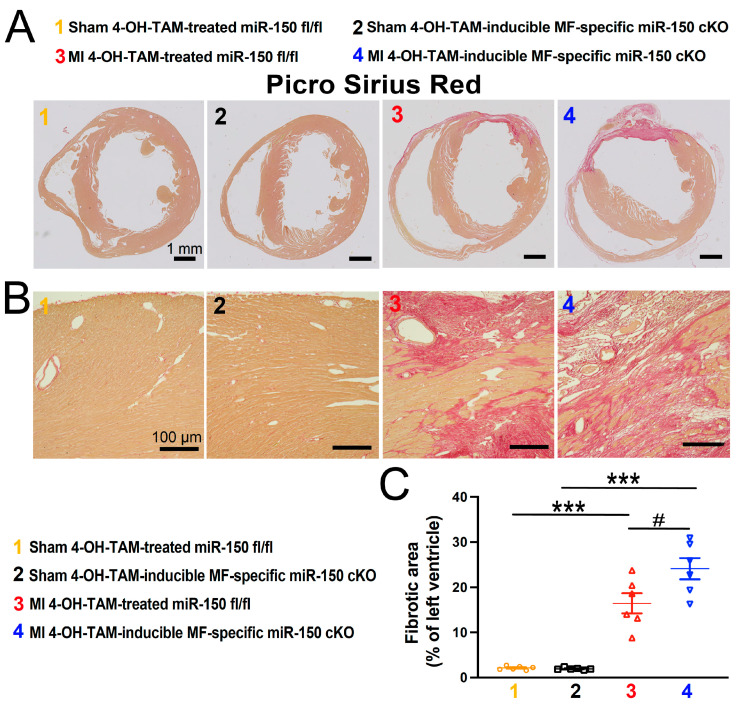
Inducible myofibroblast-specific miR-150 ablation in adult mice worsens cardiac fibrosis following chronic myocardial infarction. Representative Picro Sirius Red staining (**A**,**B**) in heart sections from the four groups at 4 weeks after MI and fibrosis quantification (**C**) in whole left ventricles. Fibrosis histology images from whole heart longitudinal sections ((**A**): Scale bars: 1 mm) and zoomed in images of the peri-ischemic border area ((**B**): Scale bars: 100 μm). N = 5–6 per group. Data are shown as the mean ± SEM. Two-way ANOVA with Tukey’s multiple comparison test. *** *p* < 0.001 vs. sham of same genotype; ^#^ *p* < 0.05 vs. MI 4-OH-TAM-treated miR-150 ^fl/fl^.

In our investigation of fibrosis post-MI, we employed Masson’s Trichrome staining and Picro Sirius Red staining to assess fibrotic areas in the hearts at 4 weeks after MI. We found small regions of fibrosis in the 4-OH-TAM-treated miR-150 ^fl/fl^ hearts, whereas hearts from the 4-OH-TAM-inducible miR-150 cKO mice exhibited significantly larger fibrotic regions (Figure 5 and Figure 6).

**Figure 7:**
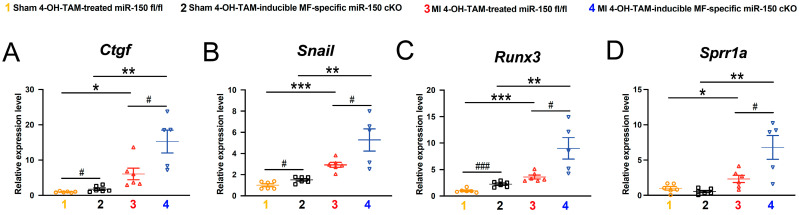
Selective loss of miR-150 in adult myofibroblasts activates the cardiac expression of profibrotic genes post-MI. QRT-PCR analysis of profibrotic *Ctgf* (**A**), *Snail* (**B**), *Runx3* (**C**), or *Sprr1a* (**D**) expression in ischemic areas from 4-OH-TAM-treated miR-150 ^fl/fl^ and 4-OH-TAM-inducible miR-150 cKO mouse left ventricles at 4 weeks after MI. Data are presented as the fold induction of gene expression normalized to *Gapdh*. N = 5–6 per group. Data are shown as the mean ± SEM. Two-way ANOVA with Tukey’s multiple comparison test. * *p* < 0.05, ** *p* < 0.01, or *** *p* < 0.001 vs. sham of same genotype; ^#^ *p* < 0.05 or ^###^ *p* < 0.001 vs. 4-OH-TAM-treated miR-150 ^fl/fl^.

Consistently, mRNA levels of fibrotic *Ctgf*, *Snail*, and *Runx3* are elevated in 4-OH-TAM-inducible miR-150 cKO hearts compared to controls (Figure 7A–C). Moreover, the expression of fibrotic *Sprr1a*, identified as a direct target of miR-150 in ischemic mouse hearts and primary adult human CFs [18], is significantly upregulated in 4-OH-TAM-inducible miR-150 cKO hearts at 4 weeks after MI (Figure 7D). Our current findings collectively demonstrate, for the first time, that the selective knockdown of miR-150 in adult MFs significantly exacerbates various cardiac abnormalities during post-ischemic remodeling.

## 4. Discussion

This study identifies miR-150, selectively expressed in MFs, as a critical protective mediator against ischemic injury-induced cardiac dysfunction and fibrosis. Adult mice with a selective deficiency of miR-150 in MFs revealed a heightened sensitivity to MI, characterized by increased damage, apoptosis, and fibrosis as well as increased expression of inflammatory genes in the heart, alongside impaired left ventricular function. Mechanistically, our current study shows that ischemic hearts lacking miR-150 in MFs display significantly elevated expression of fibrotic genes, including *Sprr1a*, *Ctgf*, *Snail*, and *Runx3*. Our previous mechanistic studies also reported that miR-150 directly and functionally inhibited multiple profibrotic proteins, including HOXA4 and SPRR1A, such that the increased fibrotic markers in adult mice or primary human CFs lacking miR-150 led to sustained activation of CFs, increased MF density, and exacerbated cardiac fibrosis during ischemia [17,18].

It is worth noting our prior study, which showed that systemic miR-150 KO mouse hearts exhibited neutrophil infiltration and CM apoptosis, without affecting T cell infiltration at 1 day after MI. The capillary density or the expression of endothelial cell markers were comparable between the WT and miR-150 KO groups at 8 weeks post-MI, and miR-150 O/E blocked ischemic mouse cardiac endothelial cell apoptosis in vitro [13]. Conversely, another group reported that systemic O/E of miR-150 in mice by AgomiR injection reduced myocardial fibrosis and protected their hearts from acute MI through decreasing monocyte migration via targeting CXCR4 [25]. This prior study suggests that miR-150 suppresses a proinflammatory phenotype in MFs and that miR-150 in peripheral blood can regulate cardiac fibrosis. Moreover, cardiac-specific O/E of miR-150 attenuated cardiac fibrosis, hypertrophy, and dysfunction induced by TAC [15]. Studies also showed that TGF-β/Smad signaling inhibited the expression of miR-150 [33,34], and that miR-150 could repress TGF-β in rat hearts in vivo and cultured CFs [35]. MMPs and TIMPs are crucial in the degradation of the extracellular matrix. Additional prior studies have reported that miR-150 suppressed MMP-2, MMP-9, MMP-13, and ADAMTS-5 [36,37], while activating TIMP-2 [38]. These findings collectively suggest an antifibrotic role of miR-150. However, despite these insights, our understanding of the overall actions of miR-150 remained incomplete, partly due to the lack of definitive studies using appropriate mouse models to address its cell type-specific actions. Previously, we showed that CM-restricted miR-150 cKO mice exhibited exacerbated maladaptive remodeling post-MI [14]. 4-OH-TAM-inducible Cre recombinase from the promoter of the mouse *Postn* in Postn-MerCreMer mice, which we use in the current study, was reported to be expressed progressively and exclusively in activated MFs after injury [27,28,29]. In this study, we initially injected 4-OH-TAM immediately after MI and then injected for an additional 4 days as previously validated [27,28,29]. We were able to observe the significant knockdown of miR-150 in our 4-OH-TAM-inducible MF-specific miR-150 cKO mice compared to three controls at 4 weeks after MI (Figure 1C). Using this novel inducible mouse model, we discovered a defining role of miR-150 specifically in adult MFs under in vivo conditions.

Our previous research highlighted that miR-150 blocked proliferation, migration, and transformation of primary adult human CFs in vitro as well as cardiac fibrosis following ischemic stress in part by repressing profibrotic markers, including ACTA2, COL1A1, COL3A1, COL4A1, COL8A1, CTGF, HOXA4, POSTN, SMAD2, SMAD3, SRF, SPRR1A, and TGFB1 [17,18]. Our current data (Figure 7) also suggest that miR-150 loss in adult MFs activates profibrotic genes, including *Ctgf*, *Snail*, *Runx3*, and *Sprr1a*. All of our findings thus indicate that the inhibition of multiple fibrotic genes is a plausible downstream mechanism by which MF-derived miR-150 regulates the response to MI.

Our prior transcriptomic profiling, filtering, validation, and mechanistic studies identified HOXA4 and SPRR1A as major direct targets of protective actions mediated by miR-150 in ischemic adult mouse hearts and primary human CFs [17,18]. Additionally, we reported that HOXA4 and SPRR1A exhibited profibrotic and maladaptive effects in mouse models of MI [17,18]. Given our previous findings that fibrotic HOXA4 and SPRR1A were crucial direct and functional targets of miR-150 [17,18], circulating miR-150 levels in patients with MI can, thus, be used to guide future treatment strategies by modulating HOXA4 and SPRR1A.

### Study Limitation

Although we showed the significance of miR-150 expression in adult MFs as a crucial negative modulator of MI, it is plausible that its expression in other cardiac cell types also plays important roles. This possibility is supported by our previous findings on CM-specific cKO mice, which indicated a role for miR-150 in CMs as well [14]. Understanding how the different sources of miR-150 work together (via paracrine fashion) or independently (via autocrine fashion) is of importance and is beyond the scope of the current study. Moreover, RNA-FISH and protein-RNA double labeling studies would be needed to confirm miR-150 knockdown as well as increased levels of fibrotic and inflammatory targets of miR-150 in MFs of our 4-OH-TAM-inducible MF-specific miR-150 cKO mouse hearts post-MI.

Despite an excessive fibrotic response over the first week after MI induction, further immunohistochemical assessments and gene expression studies at earlier time points than 4 weeks after MI need to be conducted to gain a comprehensive understanding of the sequence of events. For example, dying cell types and the status of CMs, inflammation, and vascularization at multiple time points post-MI in the context of miR-150 deletion in MFs remain elusive. Moreover, our 4-OH-TAM-inducible MF-specific cKO mouse model has limitations. As compared to 4-OH-TAM-treated miR-150^fl/fl^ hearts, we found that 4-OH-TAM-treated MF-specific miR-150 cKO hearts have increased cleaved-caspase 3-positive cells as well as increased expression of apoptotic *Bak1* (Figure 4A–C) and fibrotic markers such as *Ctgf*, *Snail*, and *Runx3* at baseline (Figure 7A–C), indicating that miR-150 may be deleted in 4-OH-TAM-treated MF-specific miR-150 cKO without MI, though to a lesser extent. This notion is supported by our previous studies, reporting that miR-150 KO hearts had increased apoptotic markers such as *Bax, p53,* and *Bak1* as well as increased fibrotic *Col1a1* at baseline and that apoptotic *P2x7r* (a direct target of miR-150), was increased in miR-150 KO sham hearts having normal function [13,18]. Finally, a constitutive cKO mouse model where miR-150 knockdown in fibroblasts is more efficiently achieved using *Postn*-Cre mice [39,40] needs to be generated to appropriately examine fibroblast roles in inflammatory and wound healing responses as well as acute MI responses (e.g., acute infarct size and acute cardiac cell death). To adequately investigate whether miR-150 is involved in fibroblast activation, *Tcf21*-Cre-ER ^T2^ mice need to breed with our miR-150 ^fl/fl^ mice. Our current results, which show the role of miR-150 in adult MFs during chronic MI, will fuel future studies to make these new miR-150 cKO mouse models.

## 5. Conclusions

Using a novel inducible cKO mouse model, our study indicates that miR-150, specifically in MFs, protects the heart from ischemic injury in adult mice. Interestingly, a previous study demonstrated that systemic O/E of miR-150 protected against acute MI in mice by suppressing monocyte migration [25]. Moreover, our previous research showed that a CM-specific loss of miR-150 exacerbated cardiac dysfunction and maladaptive cardiac remodeling by suppressing CM apoptosis during acute MI [14]. Although these prior studies revealed the roles of miR-150 in monocyte recruitment and CM function after acute ischemic injury, our current MF-specific cKO studies delineate, for the first time, the specific actions of miR-150 in adult MFs during chronic MI. Given that downregulation of miR-150 is implicated in other forms of heart disease as well [21,41,42,43], the beneficial role of miR-150 in MFs may have broad applicability across diverse stress settings. Therefore, strategies aimed at boosting miR-150 levels, such as using carvedilol or miR-150 mimic-based O/E could represent attractive adjunctive approaches to enhance therapeutic benefits.

## Data Availability

All data generated or analyzed during this study are included in this article.
